# Characterization Challenges of Self-Assembled Polymer-SPIONs Nanoparticles: Benefits of Orthogonal Methods

**DOI:** 10.3390/ijms232416124

**Published:** 2022-12-17

**Authors:** Cintia Marques, Lionel Maurizi, Gerrit Borchard, Olivier Jordan

**Affiliations:** 1 Institute of Pharmaceutical Sciences of Western Switzerland, University of Geneva, 1 Rue Michel Servet, 1211 Geneva, Switzerland; 2 Section of Pharmaceutical Sciences, University of Geneva, 1 Rue Michel Servet, 1211 Geneva, Switzerland; 3 Laboratoire Interdisciplinaire Carnot de Bourgogne (ICB), UMR 6303 CNRS—Université Bourgogne Franche-Comté, BP 47870, CEDEX, 21000 Dijon, France

**Keywords:** characterization, polydispersity, orthogonal characterization techniques, SPIONs, dynamic light scattering, nanoparticle tracking analysis, tunable resistive pulse sensing, asymmetric flow field flow fractionation

## Abstract

Size and zeta potential are critical physicochemical properties of nanoparticles (NPs), influencing their biological activity and safety profile. These are essential for further industrial upscale and clinical success. However, the characterization of polydisperse, non-spherical NPs is a challenge for traditional characterization techniques (ex., dynamic light scattering (DLS)). In this paper, superparamagnetic iron oxide nanoparticles (SPIONs) were coated with polyvinyl alcohol (PVAL) exhibiting different terminal groups at their surface, either hydroxyl (OH), carboxyl (COOH) or amino (NH_2_) end groups. Size, zeta potential and concentration were characterized by orthogonal methods, namely, batch DLS, nanoparticle tracking analysis (NTA), tunable resistive pulse sensing (TRPS), transmission electron microscopy (TEM), asymmetric flow field flow fractionation (AF4) coupled to multi-angle light scattering (MALS), UV–Visible and online DLS. Finally, coated SPIONs were incubated with albumin, and size changes were monitored by AF4-MALS-UV-DLS. NTA showed the biggest mean sizes, even though DLS PVAL-COOH SPION graphs presented aggregates in the micrometer range. TRPS detected more NPs in suspension than NTA. Finally, AF4-MALS-UV-DLS could successfully resolve the different sizes of the coated SPION suspensions. The results highlight the importance of combining techniques with different principles for NPs characterization. The advantages and limitations of each method are discussed here.

## 1. Introduction

The characterization of nanomedicines is a current topic of discussion in the literature due to the complexity of these systems [1,2,3]. Germain et al. [4] stressed the importance of preclinical characterization and regulatory uniformization as key points to increase the clinical success rate. In the same context, the REFINE white paper (2019) referred to the importance of regulatory practice harmonization at an international level [5]. Among others, the white paper refers to the challenge of standardized characterization methods, since those are often not suitable to assess different types of nanomedicines.

Recently, the FDA published their guidance on “Drug products, including biological products, that contain nanomaterials” [6], establishing the attributes for nanomaterials. There, the average particle size, particle size distribution (PSD), shape and morphology, surface charge and concentration, among others, are mentioned as mandatory attributes to be determined [6]. Size is an important critical quality attribute (CQA) of nanomedicines, since it establishes their shelf life, and it is an early design indicator for in vivo performance [7]. Moreover, size influences the biological activity and the safety profile of nanoparticles, so its determination and control is essential for further industrial upscale [8] and clinical success. Therefore, accuracy, reliability, reproducibility and robustness [3,7,8] within and between characterization techniques are important for the success of nanomedicines.

The method of size measurement differs between techniques, so comparing values obtained through different approaches is challenging [9]. First, we can divide the measurements into two big categories: size in suspension and in the dry state. The first measure is usually mentioned in terms of the hydrodynamic size, being the most commonly mentioned particle diameter, reflecting the size of the particles in suspension, including coatings and surface modifications [8].

Dynamic light scattering is the most used technique to report size due to its relative simplicity [10]. The ISO 22412 (2017) standardizes DLS measurements and defines the z-average as a “scattered light intensity-weighted harmonic mean particle diameter”, providing an average information about the sample. However, since the z-average is weighted by the scattered intensity, it is frequently biased towards bigger nanoparticles [10,11,12,13]. Similar to DLS, a nanoparticle tracking analysis measures the hydrodynamic diameter of NPs by measuring the diffusion coefficient [14]. Nonetheless, NTA measures individual particles, reporting a number–size weight, thereby reducing the bias towards larger aggregates. Therefore, NTA has been considered an alternative to classic DLS [8,9,14]. For example, Kim et al. [9] confirmed the correlation of hydrodynamic sizes of polystyrene NPs obtained by DLS and NTA, even though the results are, respectively, intensity-based and number-based [9]. Moreover, they found that native number-weighted NTA sizes and intensity-weighted DLS sizes have a better correspondence than converted values [9].

Tunable resistive pulse sensing is a particle-by-particle measuring technique based on the impedance caused by a particle passing through a nanopore. After calibration, both the size and zeta potential can be measured, improving the traceability but making it a time-consuming process. Moreover, TRPS measures individual NPs in the same way as NTA; hence, they are common techniques to measure NP concentrations [7].

More recently, asymmetric flow field flow fractionation, a chromatographic technique, drew attention, since it can be combined with several detectors, such as multi-angle light scattering, DLS, UV–Vis and the refractive index (RI) [15]. The combination of techniques offers a complete sample characterization after separation according to size, in addition to concentration (UV–Vis or RI) and hydrodynamic size (online DLS) determination [7,16]. By coupling DLS with AF4, it is possible to analyze different size populations, overcoming the bias issue mentioned above [7].

While those methods measure the particle size in suspension, dry state size techniques, such as electron microscopy (TEM and SEM), offer more information about the NP shape, size and morphology through direct imaging. However, sample preparation can create artifacts throughout the drying process and/or due to the staining required by some materials to render them electro-dense [7]. Plus, a micrograph analysis might be time-consuming and does not allow for the calculation of polydispersity [17].

Lastly, to obtain accurate size information, it is essential to select orthogonal analytical techniques [2,5,12,18]. To do so, it is important to consider the method suitability and to use complementary methods to address technique-related differences [6,7,18].

Superparamagnetic iron oxide nanoparticles (SPIONs) are biocompatible nanoparticles of controllable shapes and sizes and scale-up manufacturing capability [19]. Magnetic nanoparticles can be used for magnetic resonance imaging diagnostics, magnetic hyperthermia treatment and targeted drug delivery [19,20,21]. SPIONs tend to form fractal aggregates in suspension [22,23]. Thus, when coated with polymers, the shape of coated SPIONs might not be spherical. The size characterization of irregular, non-spherical NPs is still one of the biggest challenges for particle size determination [24], since most techniques, such as DLS, consider NPs to be spherical [14].

This work aims to characterize metal core NPs coated with different polymers, focusing on particle size and size distribution, charge and concentration. To do so, an orthogonal multi-technique characterization approach was applied. SPIONs were coated with polyvinyl alcohol (PVAL-OH) and two other copolymers, namely carboxyl-modified poly(vinyl alcohol) (PVAL-COOH) and poly(vinyl alcohol-co-vinyl amine) (PVAL-NH_2_). The coated SPIONs were characterized by batch DLS, NTA, AF4-MALS-UV-DLS, TRPS and TEM to draw conclusions on the most suitable methods for routine nanoparticle characterization. Finally, since NPs colloidal stability is commonly strongly impacted by biological fluids, we evaluated the influence of a highly abundant serum protein on the coated SPION sizes by incubating them with albumin and characterizing them by AF4-MALS-UV-DLS.

## 2. Results

### 2.1. Pre-Screening—DLS vs. NTA

#### 2.1.1. Size

**Figure 1:**
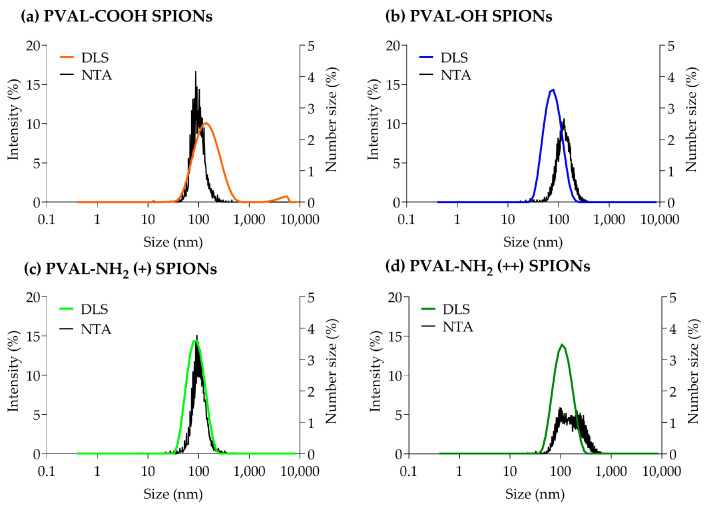
Graphs obtained from DLS and NTA particle size measurements for (**a**) PVAL-COOH SPIONs, (**b**) PVAL-OH SPIONs, (**c**) PVAL-NH_2_ SPIONs (+) and (**d**) PVAL-NH_2_ SPIONs (++). DLS size distribution was plotted as a percentage of the total intensity (left axis). NTA size distribution was plotted as a percentage of the total number of measured NPs (right axis).

Figure 1 shows that, for PVAL-OH SPIONs and for nanoparticles coated with a low and high PVAL-NH_2_/PVAL-OH ratio (PVAL-NH_2_ (+) SPIONs and PVAL-NH_2_ (++) SPIONs, respectively), the DLS intensity-based graphs are shifted to smaller sizes than NTA number-based size values. Similar conclusions can be drawn from Table 1, which reports the z-average for DLS and number-based size for NTA. The NTA tendency to report higher values can be explained by its suggested lower analytical size range (down to 10 nm for NTA vs. 0.6 nm for DLS) [11].

The PVAL-NH_2_ (++) SPIONs NTA graph does not show a sharp peak, suggesting a broader range of sizes. However, that is not supported by DLS, which shows a Gaussian distribution for the same coated SPIONs. In fact, polydispersity index (PDI) values for PVAL-OH SPIONs, PVAL-NH_2_ (+) SPIONs and PVAL-NH_2_ (++) SPIONs are around 0.1 (Table 1), revealing monodisperse populations [16]. Contrarily, PVAL-COOH SPIONs are more polydisperse, with a PDI of 0.26. The DLS graph shows the presence of big aggregates, plus a shift to higher NP sizes, when compared to the NTA graph. Nevertheless, the NTA did not show aggregates on the PVAL-COOH SPION samples.

The contradictory data obtained from the DLS and NTA measurements exposes the need of a sample analysis using high-resolution techniques, such as AF4-online DLS.

#### 2.1.2. Zeta Potential

By using PVAL with different moieties (COOH, OH and NH_2_) to coat maghemite SPIONs, it was expected to obtain, respectively, negative, neutral and positively charged coated SPIONs at pH 7.4. The zeta potential of coated SPIONs was measured in water and in phosphate-buffered saline (PBS) 0.1× (pH 7.4) by both the DLS and NTA.

Table 2 confirms that, in water, PVAL-COOH SPIONs were negatively charged (−13 mV). PVAL-OH SPIONs were almost neutral, showing low zeta potential (+10 mV), while PVAL-NH_2_ (+) SPIONs and PVAL-NH_2_ (++) SPIONs were positively charged at, respectively, +26 mV and + 36 mV. PVAL-NH_2_ (+) SPIONs were coated with PVAL-NH_2_/PVAL-OH, showing the contribution of NH_2_ moieties to the positive charge. This is further confirmed by PVAL-NH_2_ (++) SPIONs, which were more positively charged due to their higher PVAL-NH_2_/PVAL-OH ratio.

The zeta potential measured by the DLS in PBS 0.1x tends to be lower than the one measured in water. The difference was higher for the PVAL-NH_2_ (+) SPIONs and PVAL-NH_2_ (++) SPIONs charged, respectively, at +0.5 mV and + 12 mV in PBS 0.1x. This is suggested to be due to the partial neutralization of the charges of the NH_2_ moieties of the PVAL-NH_2_ polymer by PBS phosphate ions.

The zeta potential was also determined with the NTA. While PVAL-NH_2_ (+) SPIONs were slightly positive (+0.5 mV) according to the DLS, they had negative zeta potential according to the NTA (−13 mV). The difference was higher for PVAL-NH_2_ (++) SPIONs, which had a charge of +12 mV according to the DLS but a charge of −34 mV according to the NTA. Regarding the PVAL-OH SPIONs and PVAL-COOH SPIONs systems in PBS 0.1x, the zeta potential obtained from the DLS was about 10 mV higher than the one obtained from the NTA. To further explore this phenomenon, the zeta potential obtained was measured for the different coated PVAL-SPIONs in water with the NTA (Table 2). The charge in water is similar between the DLS and NTA and independent of the NP dilution, suggesting that the differences in zeta potential for NPs diluted in PBS might be related to the phosphate present in the buffer.

### 2.2. Analysis of PSD with High-Resolution Techniques

#### 2.2.1. Resolve PSD: AF4-UV-MALS-Online DLS

**Figure 2:**
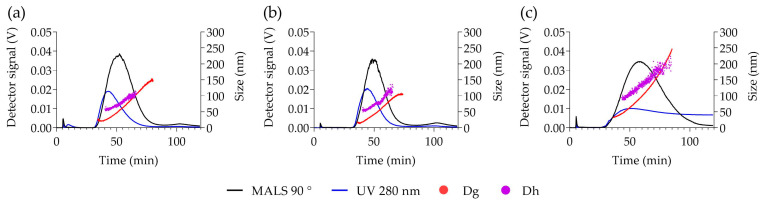
AF4-UV-MALS-DLS elution profiles of (**a**) PVAL-COOH SPIONs, (**b**) PVAL-OH SPIONs and (**c**) PVAL-NH_2_ SPIONs (+). Scattered light from MALS at 90° (black) values are plotted on the left axis. Both Dg (red) and Dh (blue) sizes are plotted on the left axis. The Dg was calculated by fitting the MALS 9 angles data with the random coil model.

The AF4-MALS-DLS fractograms are reported in Figure 2. The elution peaks were detected between 35 and 80 min for PVAL-OH SPIONs and PVAL-COOH SPIONs, and between 35 min and 110 min for PVAL-NH_2_ (+) SPIONs. UV detection identified a small shoulder for PVAL-COOH SPIONs at 10 min, which might correspond to the free polymer fraction.

The upward tendency for the diameter of gyration (Dg) and for the hydrodynamic diameter (Dh) revealed nanoparticles with a large range of sizes. PVAL-COOH SPIONs had NPs with Dg values ranging from 27 to 150 nm, PVAL-OH SPIONs NPs displayed a 17–105 nm range, and PVAL-NH_2_ (+) SPIONs revealed NPs between 27 and 250 nm. As for the Dh, PVAL-COOH SPIONs, PVAL-OH SPIONs and PVAL-NH_2_ (+) SPIONs showed size ranges of 54–106 nm, 50–125 nm and 88–205 nm, respectively. Values for Dh were higher than for Dg during the elution, except for PVAL-NH_2_ (+) SPIONs, where the tendency inverted after 90 min of elution. No data was obtained for the more positively charged PVAL-NH_2_ (++) SPIONs, due to their high interaction with the amphiphilic regenerated cellulose membrane. Focusing on the average sizes obtained from MALS and online DLS measurements described in Table 3, the Dg was lower than the Dh for PVAL-COOH SPIONs (79 < 97 nm) and for PVAL-OH SPIONs (75 < 88 nm), but the opposite tendency is observed for PVAL-NH_2_ (+) SPIONs (151 > 129 nm). Consequently, regarding the shape factor (ratio Dg/Dh), PVAL-COOH SPIONs, PVAL-OH SPIONs and PVAL-NH_2_ (+) SPIONs had shape factors of 0.92, 0.77 and 1.04, respectively.

#### 2.2.2. NPs Size and Concentration: NTA vs. TRPS

Both the NTA and TRPS are particle-by-particle techniques, the reason why they are often compared in the literature [25,26].

**Figure 3:**
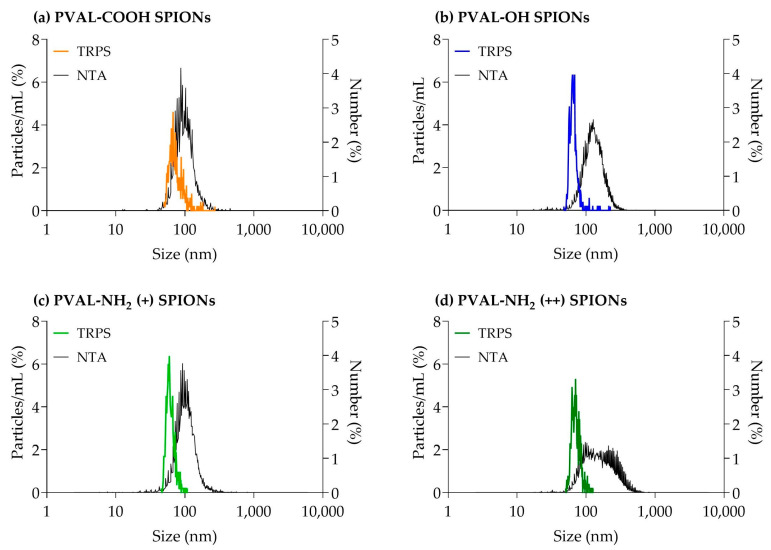
Graphs obtained from TRPS and NTA particle size measurements for (**a**) PVAL-COOH SPIONs, (**b**) PVAL-OH SPIONs, (**c**) PVAL-NH_2_ SPIONs (+) and (**d**) PVAL-NH_2_ SPIONs (++). TRPS size distribution was plotted as a percentage of the total particles/mL (left axis). The NTA size distribution was plotted as a percentage of the total number of measured NPs (right axis).

The mean number-based sizes measured by TRPS for PVAL-COOH SPIONs, PVAL-OH SPIONs, PVAL-NH_2_ (+) SPIONs and PVAL-NH_2_ (++) SPIONs were, respectively, 79 nm, 68 nm, 63 nm and 74 nm (Table 4 and Figure 3). All these were 0.56×, 0.64×, 0.56× and 0.38× smaller than the average size obtained by the NTA, correspondingly. The same tendency was observed for the concentration of nanoparticles in suspension, since TRPS detected NP concentrations between 0.27× and 0.02× lower than NTA.

#### 2.2.3. Morphology: TEM

**Figure 4:**
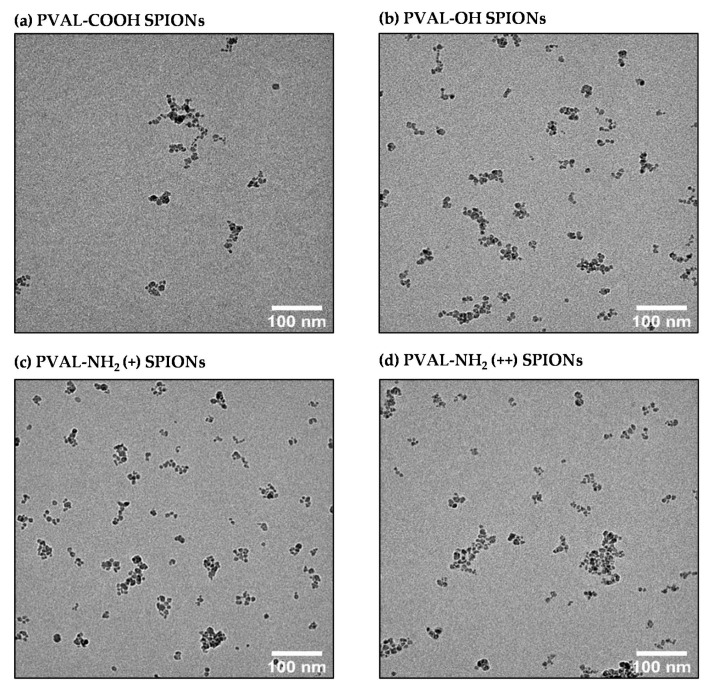
TEM micrographs of (**a**) PVAL-COOH SPIONs, (**b**) PVAL-OH SPIONs, (**c**) PVAL-NH_2_ (+) SPIONs and (**d**) PVAL-NH_2_ (++) SPIONs.

TEM is a valuable technique to visualize the topography, shape and NP distribution [9]. Some papers apply it to a number-based size determination [16]. However, this depends on the electrodensity of the material. Since the PVAL is not electrodense, the micrographs (Figure 4) only allowed the visualization of the core composed by several SPIONs. Hence, the measured size will be underestimated. Moreover, the method is user-dependent, since only particles on the small chosen area of the grid will be measured (nonrepresentative sample) [16]. Another drawback is the sample preparation, since the drying process can deform NPs, leading to false readings [27].

### 2.3. Coated SPIONs Interaction with Bovine Serum Albumin (BSA)

**Figure 5:**
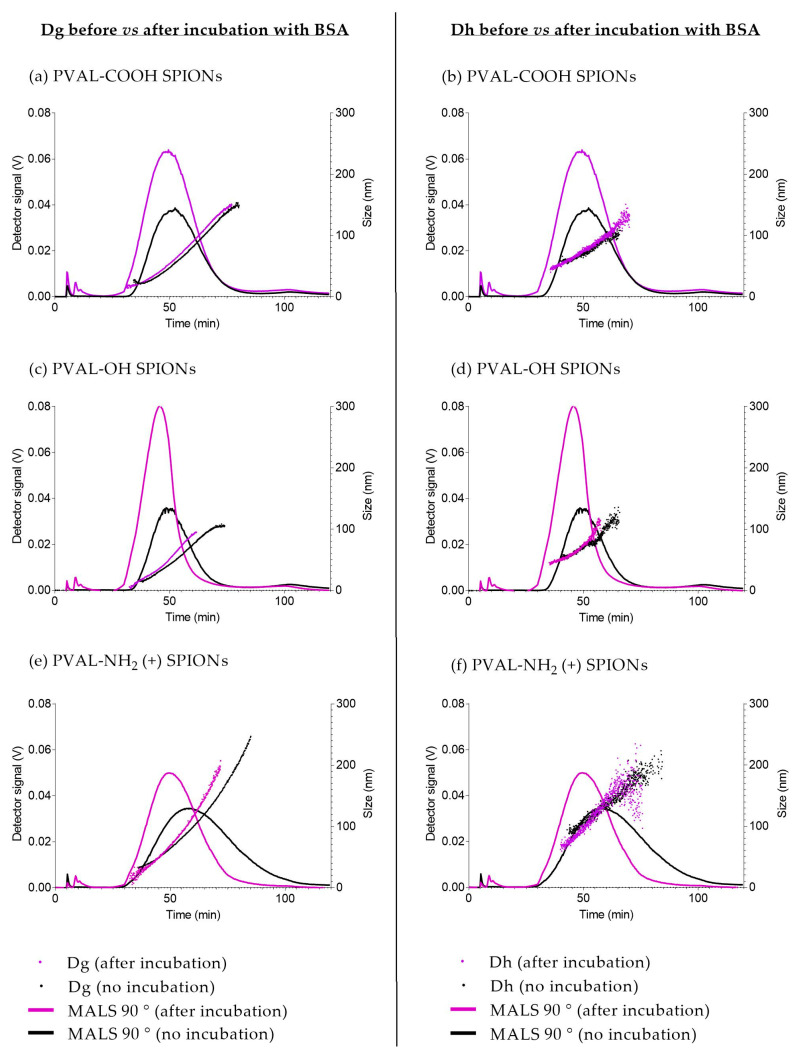
AF4-MALS-online DLS elution profiles before (black) and after (purple) incubation with BSA. (**a**,**b**) PVAL-COOH SPIONs; (**c**,**d**) PVAL-OH SPIONs; (**e**,**f**) PVAL-NH_2_ SPIONs (+). Fractograms correspond to a MALS 90° detector (plotted on left axis). (**a**,**c**,**e**) Dg obtained. (**b**,**d**,**f**) The obtained Dh. A free BSA peak was detected at 10 min.

To study NPs stability after the interaction with serum proteins, the size of coated SPIONs after incubation with BSA was measured by AF4-MALS-UV-DLS. The elution method was developed to resolve two peaks: one peak of unbound BSA (10 min) and one peak of BSA adsorbed onto coated-SPIONs (after 30 min). Following incubation with BSA (Figure 5, purple graphs), coated SPIONs eluted earlier than the nonincubated ones—about 5 min earlier for PVAL-COOH and SPIONs, about 9 min earlier for PVAL-OH SPIONs and about 4 min earlier for PVAL-NH_2_ (+) SPIONs. Right shifts on MALS elution peaks are usually related to smaller NP sizes, which is supported by the data displayed in Table 5. For PVAL-COOH SPIONs, the Dg after incubation with BSA was 5.69 nm smaller than the Dg before incubation. The same tendency was found for PVAL-OH SPIONs and PVAL-NH_2_ (+) SPIONs, whose Dg were, respectively, 29.05 nm and 61.46 nm smaller after incubation. This was also confirmed by online DLS, where the Dh decreased by 23.6 nm, 18.03 nm and 12.10 nm after incubation for PVAL-COOH SPIONs, PVAL-OH SPIONs and PVAL-NH_2_ (+) SPIONs, respectively.

## 3. Discussion

Due to its simplicity, DLS is the most used technique to report particle size. However, measurements are frequently biased towards bigger NPs due to its sensitivity to large particles or aggregates [8,14]. This was not the case in this study. For PVAL-COOH SPIONs, even though there were some aggregates present in the micrometer range (Figure 1a), the z-average (130 nm) was smaller than the peak 1 mean (153 nm), suggesting the presence of numerous small NPs [28]. The same tendency was found for PVAL-OH SPIONs, PVAL-NH_2_ (+) SPIONs and PVAL-NH_2_ SPIONs (++), where the z-average was smaller than the peak 1 mean (Table 1).

Concerning the comparison of the results between orthogonal techniques NTA and DLS, the PVAL-COOH SPIONs mean number-based size (140 nm) was higher than the z-average (130 nm). That is probably due to the lower sensitivity of the technique, which does not detect smaller NP populations [8]. Plus, since NTA measurements are number-based, the results might suggest that NPs with 140 nm, 107 nm, 112 nm and 195 nm are the predominant sizes in the NP population for PVAL-COOH SPIONs, PVAL-OH SPIONs, PVAL-NH_2_ (+) SPIONs and PVAL-NH_2_ (++) SPIONs, respectively [8].

According to the supplier, the NTA can detect NPs down to a size of 10 nm, while TRPS can detect NPs up to 40 nm. Therefore, in the presence of small NPs (as suggested by batch DLS measurements), it would be expected that the TRPS average size would be higher than the one measured by the NTA. However, the TRPS average size obtained was between 0.56× and 0.38× the sizes obtained by the NTA (Table 4). The same tendency was observed in Figure 3, where TRPS peaks were shifted towards smaller NPs than the NTA. TRPS follows the same tendency as AF4, whose UV graphs revealed higher intensities for smaller NPs (Figure S1). Thus, TRPS was able to resolve the polydisperse profile of coated SPIONs [29].

Regarding the NP concentration, Akers et al. [25] found that NTA detected higher concentrations of extracellular vesicles than TRPS for particles of sizes < 150 nm. The same trend is followed by the measurements of coated SPIONs presented in Table 4, where it is possible to conclude that NTA tends to detect larger amounts of NPs.

Concerning the different sizes, NP concentrations and zeta potential, obtained by the DLS, NTA and TRPS, it is important to consider the sample concentration measured by each technique. While samples measured by DLS and TRPS had concentrations in the mg Fe/mL range, the NTA samples were diluted to concentrations of μg Fe/mL (Table 1 and Table 4). Hence, the high dilution suffered by the latter might have led to the selection of the NP population, as well as it might have changed the NP structure and their physicochemical characteristics. This is supported by the zeta potential results (Table 2), since the values obtained for the PVAL-NH_2_ (+) and PVAL-NH_2_ (++)-coated SPIONs were negative, probably due to the higher amount of PBS phosphate ions per surface.

Although DLS reports sizes based on intensity, it can be converted into a number-based size by application of the Mie theory [30], a mathematical operation based on spherical and monodisperse NPs. PVAL-OH SPIONs, PVAL-NH_2_ (+) SPIONs and PVAL-NH_2_ (++) SPIONs have a PDI of around 0.1; thus, the NP populations may be considered to be monodisperse. However, the number-based size differs from the z-average (Table 1), suggesting the presence of larger aggregates, while TEM micrographs suggest non-spherical NPs (Figure 4). Instead, SPION cores organize as fractal aggregates onto which the polymers will adsorb. Furthermore, the shape ratio (Dg/Dh) also gives insights into the particle shape. The hydrodynamic radius corresponds to the radius of a hard sphere that diffuses at the same rate as that solute, while the gyration radius corresponds to the root mean square distance between each point in the object and its center of mass [31]. In general, NPs are spherical for values below 1 and with elongated structures for values equal or superior to 1.5 [32,33]. Hence, and in contrast to the other techniques, the obtained shape ratios show that PVAL-COOH SPIONs and PVAL-NH_2_ (+) SPIONs were hollow spheres, while PVAL-OH SPIONs were homogeneous spheres (Table 2) [32].

The analysis of PSD characterization is one of the biggest challenges, which is due not only by the technique itself but also by the instrument sensitivity, media, dilution, temperature, etc. PDI is a typical parameter applied for PSD classification by DLS. According to the ISO 22412:2017, a monodisperse sample of spherical particles should have a PDI below 0.07. However, in the literature, while some authors classified a sample as monodisperse for values below 0.1 [9], others claimed that polydisperse samples have a PDI exceeding 0.4 [8]. Consequently, PVAL-COOH SPIONs with a PDI of 0.26 (Table 1) can have an ambiguous classification. The graphs obtained from the different techniques suggest that, as discussed above, there are aggregates in the micrometer range for PVAL-COOH SPIONs. However, overall, that was not a relevant population, as they were not detected either by the NTA (Figure 1a) or by the AF4-MALS-UV-DLS fractogram (Figure 2a).

For an accurate analysis of the polydisperse samples, it is important to separate populations prior to DLS measurements [30]. AF4 coupling to DLS resolved the polydisperse samples, as shown in Figure 2, demonstrating the efficient separation of complex samples by a gradient elution [16]. Some authors claimed that AF4 has a lower resolution for small NPs [16,27], but the peak maximum for UV graphs was shifted more towards smaller sizes than the MALS graphs (Figure S1), which can lead to an overestimation of the presence of smaller NPs [16]. In addition, aggregates in the micrometer range detected for PVAL-COOH SPIONs by DLS were not visible by the AF4-online DLS analysis. This might be explained by an insufficient DLS signal from the aggregates after AF4 separation.

PVAL-NH_2_ (+) SPIONs upon AF4 elution and MALS-DLS analysis revealed bigger NPs than the ones detected by other techniques. However, it is important to note that AF4 has limited applications in positively charged NPs due to high interactions with the separation membrane. To overcome the problem, it is possible to set the mobile phase to pH 4, which renders a negative charge to the regenerated cellulose membranes [34]. Alternatively, it is also possible to condition the regenerated cellulose membrane with several injections of BSA [35]. Finally it is possible to saturate the membrane with several injections of a positive sample [16]. This work aimed to study the characteristics of NPs in relevant biological media, so the experiments were performed at pH 7.4. The membrane saturation with several injections of PVAL-NH_2_ (+) SPIONs and PVAL-NH_2_ (++) SPIONs was tested without success. In addition, BSA passivation was not sufficient to decrease the interaction of positive NPs with the regenerated cellulose membrane.

In addition to the charge, the nature of the AF4 membrane is another key factor. Since the classic regenerated cellulose membrane is covered with a hydrophobic layer, NP hydropathy plays a role in membrane interactions [16]. Therefore, PVAL-NH_2_ (+) SPIONs and PVAL-NH_2_ (++) SPIONs were injected (under the same conditions) using an amphiphilic regenerated cellulose membrane. Only with the latter membrane, it was possible to detect the PVAL-NH_2_ (+) SPIONs, though the broad peak observed in Figure 2 suggests remaining interactions. Unfortunately, it was not possible to detect PVAL-NH_2_ (++) SPIONs, due to their high positive charge. Finally, an AF4-MALS pre-calibration with the standard samples is frequently recommended, even though it is limited by the different NP interactions with the membrane [36]. BSA injections were performed as the control, and the expected peak was detected (Figure S2).

**Figure 6:**
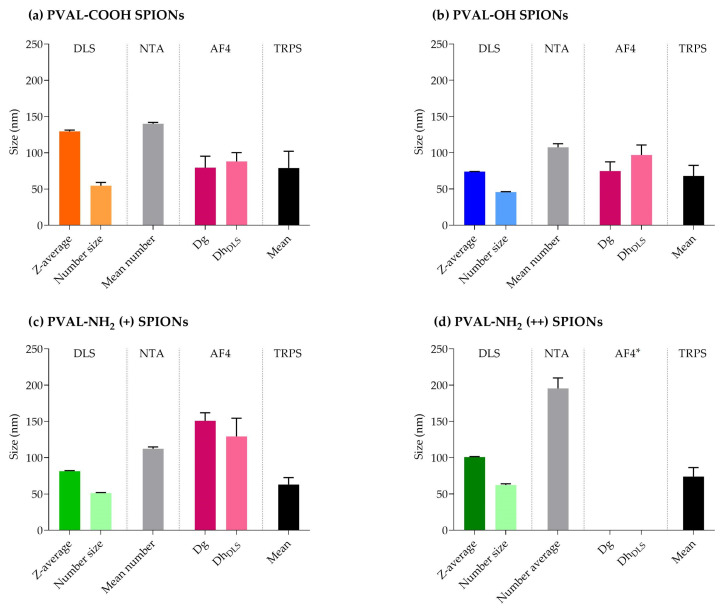
Summary of the average sizes obtained by different sizing techniques. (**a**) PVAL-COOH SPIONs, (**b**) PVAL-OH SPIONs, (**c**) PVAL-NH_2_ (+) SPIONs and (**d**) PVAL-NH_2_ (++) SPIONs. * Data not available due to the high interaction between PVA-NH_2_ (++) SPIONs and the amphiphilic regenerated cellulose membrane.

Figure 6 summarizes the main data obtained from all the sizing techniques. From there, we can conclude that:

1. NTA detected the highest NPs sizes, while TRPS displays the lowest mean values;
2. Z-average and number-based sizes (measured by DLS) present different values for all the coated SPIONs;
3. AF4-MALS-UV-DLS has limited the application to positively charged NPs;
4. Each technique seems to reveal specific information about the coated SPIONs, disclosing their complexity.

Finally, once NPs are injected into the bloodstream, serum proteins will adsorb onto their surface, forming the biomolecular corona. That can change physicochemical identity of NPs, leading, for example, to changes in NP size due to aggregation, disaggregation or destabilization of the particles [12,37]. CDER/FDA and the European Nanomedicine Characterization Laboratory (EU NCL) mention in recent publications [11,12] the need to study the interaction between NPs and serum to have a complete characterization. Albumin is the most abundant protein in human blood, so coated SPIONs were incubated with BSA to track changes in NPs size [11]. AF4 is widely applied to study the interaction between serum proteins and NPs since it can detect small size shifts [38]. Hence, the AF4 method was developed to be able to obtain two resolved peaks: one peak of unbound BSA (10 min) and one peak of BSA adsorbed onto coated SPIONs (after 30 min) (Figure 4). In general, incubation with proteins is expected to increase NPs size [11,39,40], but the coated SPIONs showed the opposite behavior. The decrease in BSA-coated SPIONs size suggests a stabilization of the polymer–SPIONs core (Table 5). In fact, the decrease of the shape factor for both PVAL-NH_2_ (+) SPIONs and PVAL-OH SPIONs indicated the formation of a more condensed structure due to the interactions with BSA. The latter has a pI of 4.5, meaning BSA is negatively charged at pH 7.4. According to Table 2, under these conditions, PVAL-COOH SPIONs are also negatively charged, which might explain the increase in the shape factor after incubation with BSA. Plus, the MALS peaks after incubation are sharper for PVAL-NH_2_ SPIONs, once the BSA adsorption decreases the interactions with the positively charged membrane. This behavior has been reported before by Caputo et al. [11] after the incubation of lipidots with plasma proteins.

## 4. Materials and Methods

### 4.1. Materials

Polyvinyl alcohol (PVAL-OH; Kuraray Poval 3-85) and carboxyl modified polyvinyl alcohol (PVAL-COOH; Kuraray Poval 3-86 SD) with an average molecular weight (MW) as measured by size exclusion chromatography-multiangle laser light scattering (SEC-MALS) of 26,800 g/mol and 15,460 g/mol, respectively, were supplied by Kuraray Co. Ltd. (Switzerland). Polyvinyl/Vinyl amine copolymer (PVAL-NH_2_; Selvol Ultiloc 5003) of a MW of 39,210 g/mol was supplied by Sekisui Chemical Co. Ltd. (USA), BSA (66 kDA, A9647), PBS 10× and other chemicals were purchased from Sigma-Aldrich (Buchs, Switzerland).

### 4.2. PVAL Coatings

Maghemite SPIONs (γ-Fe_2_O_3_, 159.687 g/mol) were synthesized by the coprecipitation method [41]. SPIONs coating was based on the protocol established by Sakulkhu et al. [42,43]. The aqueous polymer solutions were prepared by dissolving the powders in Milli-Q^®^ water (2% PVAL-NH_2_, 6% PVAL-OH and 6% PVAL-COOH) for 15 min (PVAL-OH and PVAL-COOH) or 1 h (PVAL-NH_2_) at 100 °C. Then, the polymer solution was filtered through a sterile filter (pore size 40 µm).

To obtain neutral and negatively charged nanoparticles (Table 6), 10 mg Fe/mL SPIONs suspension were mixed at a *v*/*v* ratio of 1:1 with 6% PVAL-OH or 6% PVAL-COOH, respectively. To produce slightly positive and highly positive SPIONs, 6% PVAL-OH and 2% PVAL-NH_2_ solutions where mixed at a molar ratio PVAL-OH/PVAL-NH_2_ of either 40 or 7, respectively. Then, this polymer solutions were also mixed with 10 mg Fe/mL SPIONs suspension at a *v*/*v* ratio of 1:1. All coated SPIONs were kept at 4 °C.

### 4.3. Batch-Mode DLS

The z-average, PDI and zeta potential were measured by DLS and Electrophoretic Light Scattering (ELS) (Zetasizer nano-ZS, Malvern Panalytical, Malvern, UK). The Zetasizer was equipped with a red 633 nm He–Ne laser, and measurements were performed at a 173° degree scattering angle. All measurements were performed at 25 °C. The refractive index (RI) and the value for the viscosity of iron oxide (2.420; 0.887 cP) were used. The laser power attenuator was adjusted automatically.

Size and zeta potential measurements were performed in triplicate (n = 3) in DTS1070 reusable cuvettes, after sample dilution (0.5 mg Fe/mL). Particle z-average, peak 1 mean, PDI and zeta potential were determined in Milli-Q^®^ water and PBS 0.1×. Data were analyzed by Malvern Instruments Zetasizer Software version 7.12.

### 4.4. NTA

The size and zeta potential were analyzed in water and PBS 0.1× (NTA; Particle Matrix ZetaView). Measurements were performed with a ZetaView^®^ Z-NTA cell in triplicate (n = 3). Samples were diluted to reach the nanoparticles count required by software (0.067 μgFe/mL of SPIONs for PVAL-COOH SPIONs and PVAL-OH SPIONs; 0.045 µgFe/mL of SPIONs for PVAL-NH_2_ (+) SPIONs; 0.011 µgFe/mL of SPIONs for PVAL-NH_2_ (++) SPIONs). Laser wavelength was 520 nm, and measurements were taken over 1 cycle at 11 positions. Zeta potential was measured on pulses at −20 V and + 20 V. Results are shown as number average ± SD for size and zeta potential.

### 4.5. AF4-UV-MALS-Online DLS

To perform size measurements, an AF2000 MultiFlow Field Flow Fractionation system (Postnova Analytics GmbH, Landsberg am Lech, Germany) was connected to (i) a Postnova 3609 multiangle light scattering (MALS) detector (9 angles: 28°, 44°, 60°, 76°, 90°, 108°, 124°, 140° and 156°); (ii) a Waters 2487 UV detector and a (iii) DLS detector (Zetasizer nano-ZS, Malvern Panalytical, Malvern, UK). Phosphate buffer pH 7.4 was used as a mobile phase, the detector flow was kept at 0.5 mL/min and the optimized elution is described in Table 7.

All coated SPIONs were diluted with PBS 0.1× to reach a concentration of 0.25 mg Fe/mL SPIONs, and 100 µL were injected into the AF4 channel (350 µm spacer, 10 kDa amphiphilic regenerated cellulose membrane). To study the coated SPIONs behavior in the presence of BSA, 0.25 mg Fe/mL of coated SPIONs were incubated with 2.9 mg/mL of BSA (1 h at 37 °C) before injection. The absorbance was monitored at 280 nm. The Dg was measured by MALS and the results fitted by applying the random coil model. The Dh was measured by DLS. Both Dg and Dh are presented as a mean value ± SD out of three independent experiments.

### 4.6. TRPS

Tunable resistive pulse sensing was used to characterize coated SPIONs based on their size and concentration. By tuning the parameters of voltage, stretch and pressure, the nanoparticles, which were suspended in an electrolyte (0.22 µm filtered PBS), were directed through nanopores. When a nanoparticle passed through the nanopore, it caused a transient blockade that was then detected by the Izon Control Suite software (Ver. 3.4.2.51). Size was measured by the blockade magnitude, and the concentration was measured by the blockade frequency. The blockade magnitude and frequency were then converted into their corresponding physical properties by calibration employing calibration particles of known size and concentration.

The coated SPIONs suspensions were diluted to 0.025 mg Fe/mL with PBS. Then, NPs were measured using NP100 nanopores (Izon Science, New Zealand). The measurements were then calibrated with calibration particles CPC 100 (100 nm mean diameter) (Izon Science, New Zealand). Final concentration was then expressed as the number of particles per mL.

### 4.7. TEM

Coated SPIONs were diluted 500× in Milli-Q^®^ water (0.01 mg Fe/mL), and 5 μL were placed onto a negatively charged copper grid (400 mesh). After 1 min, the grid was dried, and the size and shape of the magnetic nanoparticles were evaluated with a Talos L120C (120 KeV, LaB6) transmission electron microscope. The images were processed with Fiji software [44].

## 5. Conclusions

Ideally, a characterization technique would be able to inform about NPs size, charge, shape, polydispersity, concentration, particle composition and interactions with biological media. When choosing the complementary techniques, the concentration range relevant for storage and clinical application, as well as appropriate media, should be taken into consideration. Moreover, techniques should be complementary by combining a liquid sample analysis (e.g., DLS/NTA/TRPS) with an imaging technique (e.g., TEM).

DLS is still the method of election due to its simplicity and accessibility. However, this work clearly shows that this technique depicts an incomplete and biased information of NPs, raising safety and regulatory issues. Regulatory authorities should demand a second analysis with a particle-by-particle technique, such as NTA or TRPS. The AF4-MALS-UV-DLS is also very efficient in resolving NP polydispersity, but it requires method optimization, which is time-consuming. In addition, AF4 has limited applications in positively charged NPs.

It is clear that the inappropriate assessment of NPs basic characteristics from an early stage is one of the main contributors to the reduced amount of approved nanomedicines on the market. Simplicity and accessibility of techniques seem to be the main factors contributing to this problem. Thus, it is important to focus on developing structures and initiatives to share knowledge and promote the access to characterization techniques, such as the ones promoted by Nanotechnology Characterization Laboratory (United States) and EU NCL (Europe). By doing so, the scientific community will be working towards higher clinical success and nanomedicines’ cost-effectiveness.

## Figures and Tables

**Table 1:** Particle sizes obtained by DLS and NTA. All values are averaged from 3 different replicates.

System	DLS					NTA	
	Z-Average (nm)	PDI	Peak 1 Mean ^1^ (nm)	Number Size ^1^ (nm)	Concentration (mg Fe/mL)	Mean Size (nm)	Concentration (μg Fe/mL)
PVAL-COOH SPIONs	130 ± 2	0.26 ± 0.01	153 ± 16	55 ± 4	0.50	140 ± 2	0.067
PVAL-OH SPIONs	71 ± 4	0.12 ± 0.01	82 ± 16	44 ± 2	0.50	107 ± 5	0.067
PVAL-NH_2_ (+) SPIONs	81 ± 1	0.12 ± 0.00	93 ± 1	51 ± 1	0.50	112 ± 3	0.045
PVAL-NH_2_ (++) SPIONs	101 ± 17	0.14 ± 0.01	118 ± 2	62 ± 2	0.50	195 ± 14	0.011

^1^ Described in the DLS report.

**Table 2:** Zeta potential measurements by the DLS and NTA. All values are averaged from 3 different replicates.

System	DLS			NTA		
	Zeta Potential (mV)		Concentration (mg Fe/mL)	Zeta Potential (mV)		Concentration (μg Fe/mL)
	Water	PBS 0.1x		Water	PBS 0.1x	
PVAL-COOH SPIONs	−13 ± 4	−13.1 ± 0.2	0.50	−40 ± 1	−26 ± 2	0.067
PVAL-OH SPIONs	+10 ± 3	−4.0 ± 0.3	0.50	+12 ± 5	−16 ± 1	0.067
PVAL-NH_2_ (+) SPIONs	+26 ± 6	+0.5 ± 0.3	0.50	+20 ± 1	−13 ± 1	0.045
PVAL-NH_2_ (++) SPIONs	+36 ± 2	+12 ± 1	0.50	+47 ± 1	−34 ± 1	0.011

Main peak size described in the DLS report.

**Table 3:** Particle sizes obtained by MALS (Dg by random coil model) and online DLS (Dh by cumulant analysis). All values are averaged from 3 different replicates.

System	AF4-MALS (Dg)			AF4-Online DLS (Dh)			Shape Factor
	Mean (nm)	Median (nm)	Concentration (mg Fe/mL)	Mean (nm)	Median (nm)	Concentration (mg Fe/mL)	Dg/Dh
PVAL-COOH SPIONs	79 ± 16	78 ± 8	0.25	97 ± 14	87 ± 12	0.25	0.92 ± 0.26
PVAL-OH SPIONs	75 ± 13	72 ± 7	0.25	88 ± 12	93 ± 15	0.25	0.77 ± 0.025
PVAL-NH_2_ (+) SPIONs	151 ± 11	132 ± 98	0.25	129 ± 25	131 ± 36	0.25	1.04 ± 0.083
PVAL-NH_2_ (++) SPIONs	No peak detected						

**Table 4:** Particle size and concentration obtained by the NTA and TRPS. All values are averaged from 3 different replicates. The ratio TRPS/NTA calculated for the size and concentration illustrate the difference between the techniques (last 2 columns).

System	TRPS			NTA			TRPS/NTA Ratios	
	Mean Size (nm)	Concentration (NPs/mL) ^1^	Concentration (mg Fe/mL) ^2^	Mean Size (nm)	Concentration (NPs/mL) ^1^	Concentration (μg Fe/mL) ^2^	Mean Size Ratio	Concentration (NPs/mL) Ratio
PVAL-COOH SPIONs	79 ± 13	1.52 × 10^11^	0.025	140 ± 2	5.54 × 10^11^	0.067	0.56	0.27
PVAL-OH SPIONs	68 ± 15	1.80 × 10^10^	0.025	107 ± 5	1.79 × 10^11^	0.067	0.64	0.10
PVAL-NH_2_ (+) SPIONs	63 ± 10	8.70 × 10^10^	0.025	112 ± 3	1.06 × 10^12^	0.045	0.56	0.08
PVAL-NH_2_ (++) SPIONs	74 ± 13	5.34 × 10^10^	0.025	195 ± 14	2.92 × 10^12^	0.011	0.38	0.02

^1^ Concentration of NPs normalized to Fe concentration corresponding to 0.5 mg Fe/mL. ^2^ Concentration of Fe in the sample measured by the technique.

**Table 5:** Particle size obtained by MALS before and after incubation with BSA (Dg by the random coil model) and online DLS (Dh by a cumulant analysis). All values are averaged from 3 different replicates.

System	Before Incubation			After Incubation with BSA		
	Dg (nm)	Dh (nm)	Shape Factor (Dg/Dh)	Dg (nm)	Dh (nm)	Shape Factor (Dg/Dh)
PVAL-COOH SPIONs	79 ± 16	97 ± 14	0.92 ± 0.26	74 ± 5	73 ± 9	1.02 ± 0.15
PVAL-OH SPIONs	76 ± 13	88 ± 12	0.77 ± 0.03	46 ± 1	70 ± 6	0.65 ± 0.06
PVAL-NH_2_ (+) SPIONs	151 ± 11	129 ± 25	1.03 ± 0.08	89 ± 2	117 ± 8	0.77 ± 0.06
PVAL-NH_2_ (++) SPIONs	No peak detected					

**Table 6:** Summary of SPIONs coating preparation with a corresponding volume of polymer solutions.

System	Polymer Solution			Maghemite SPIONs Suspension
	6% PVAL-COOH (mL)	6% PVAL-OH (mL)	2% PVAL-NH_2_ (mL)	SPIONs 10 mg Fe/mL (mL)
PVAL-COOH SPIONs	1.0	-	-	1.0
PVAL-OH SPIONs	-	1.0	-	1.0
PVAL-NH_2_ (+) SPIONs	-	0.9	0.1	1.0
PVAL-NH_2_ (++) SPIONs	-	0.6	0.4	1.0

**Table 7:** Elution parameters for the AF4-UV-MALS measurements.

Step	Focus Flow	Tip Flow	Cross Flow		Time
	mL/min	mL/min	mL/min	Type	mL/min
Focus	3.30	0.20	3.00	-	3.0
Elution	-	3.50	3.00	Constant	25.0
	-	3.50–0.50	3.00–0.10	Exponential power 0.2	70.0
	-	0.50	0.10	Constant	20.0
Rinse	0.05	0.05	-	-	0.5

## Supplementary Materials

The following supporting information can be downloaded at: https://www.mdpi.com/article/10.3390/ijms232416124/s1.

## Abbreviations

AF4	Asymmetric Flow Field Flow Fractionation
BSA	Bovine Serum Albumin
CDER/FDA	Center for Drug Evaluation and Research/U.S. Food and Drug Administration
CQA	Critical Quality Attribute
Dg	Diameter of gyration
Dh	Hydrodynamic diameter
DLS	Dynamic Light Scattering
EU NCL	European Nanomedicine Characterization Laboratory
MALS	Multi-Angle Light Scattering
NP	Nanoparticle
NTA	Nanoparticle Tracking Analysis
PBS	Phosphate Buffered Saline
PDI	Polydispersity Index
PSD	Particle Size Distribution
PVAL-COOH	Carboxyl-modified Poly(Vinyl Alcohol)
PVAL-COOH SPIONs	Maghemite core SPIONs coated with PVAL-COOH
PVAL-NH_2_	Poly(Vinyl Alcohol-Co-Vinyl Amine)
PVAL-NH_2_ (+) SPIONs	Maghemite core SPIONs coated with low ratio PVAL-NH_2_/PVAL-OH
PVAL-NH_2_ (++) SPIONs	Maghemite core SPIONs coated with high ratio PVAL-NH_2_/PVAL-OH
PVAL-OH	Polyvinyl Alcohol
PVAL-OH SPIONs	Maghemite core SPIONs coated with PVAL-OH
SPIONs	Superparamagnetic Iron Oxide Nanoparticles
TEM	Transmission Electron Microscopy
TRPS	Tunable Resistive Pulse Sensing
