# Supplementary material for: Characterization Challenges of Self-Assembled Polymer-SPIONs Nanoparticles: Benefits of Orthogonal Methods
Source: Int J Mol Sci. 2022 Dec 17;23(24):16124. doi: 10.3390/ijms232416124 (PMC9786186; doi:10.3390/ijms232416124)
Supplement: Supplementary file 1 [file ijms-23-16124-s001.zip › ijms-2087697-supplementary.pdf]

Characterization challenges of self-assembled polymer-SPIONs nanoparticles: benefits of orthogonal methods

Supplementary data

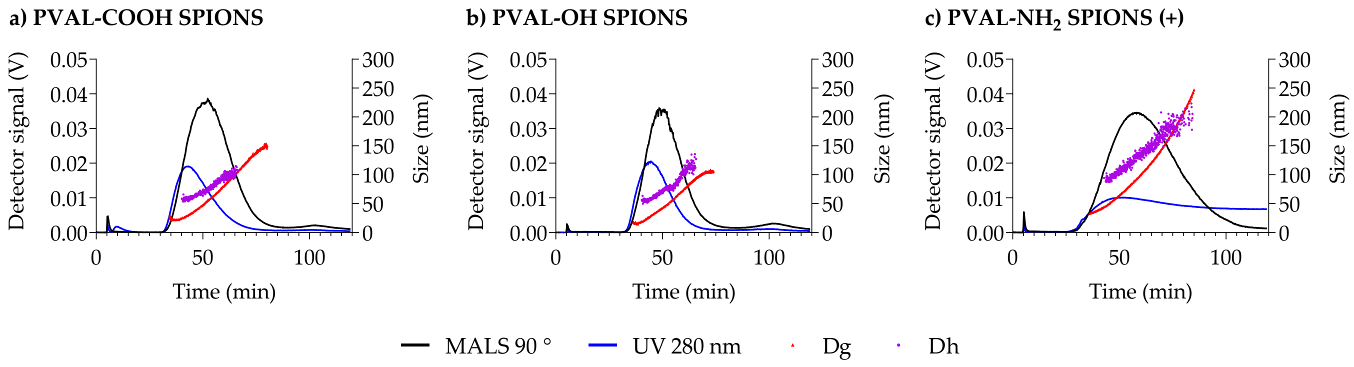

Figure S1. AF4-UV-MALS-DLS elution profiles of a) PVAL-COOH SPIONS, b) PVAL-OH SPIONS, and c) PVAL-NH_2 SPIONS (+). Scattered light from MALS at 90 ° (black) and UV absorption at 280 nm (blue) values are plotted on the left axis. Both Dg (red) and Dh (blue) sizes are plotted on the left axis. The Dg was calculated by fitting the MALS 9 angles data with the random coil model.

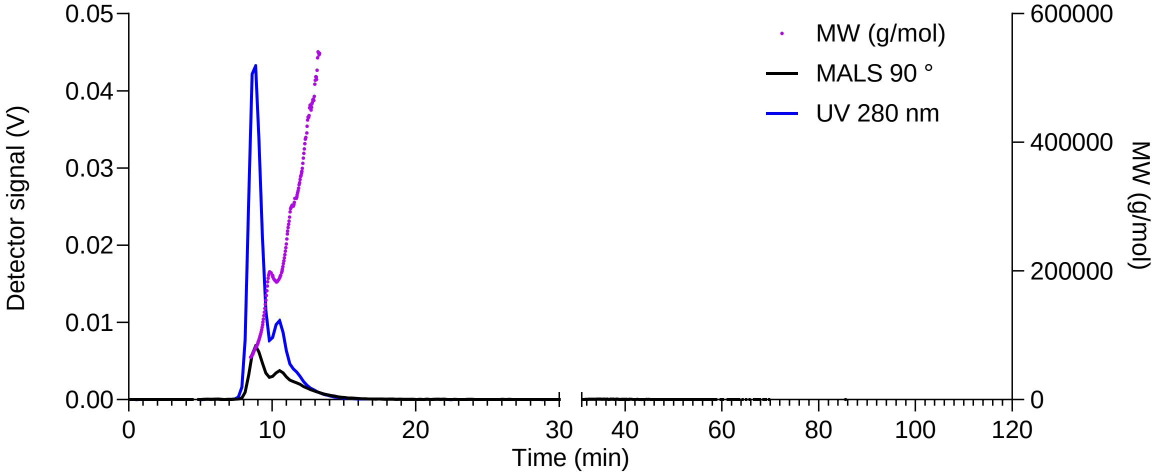

Figure S2. BSA elution profile by AF4-UV-MALS-DLS. Scattered light from MALS at 90 ° (black) and UV absorption at 280 nm (blue) values are plotted on the left axis. Molecular weight (MW) (purple) is plotted on the left axis. The MW was calculated by fitting the MALS 9 angles data with the zimm model.
